# Human Papillomavirus Infection in a Male Population Attending a Sexually Transmitted Infection Service

**DOI:** 10.1371/journal.pone.0054375

**Published:** 2013-01-23

**Authors:** Marta Elena Álvarez-Argüelles, Santiago Melón, Maria Luisa Junquera, Jose Antonio Boga, Laura Villa, Sonia Pérez-Castro, María de Oña

**Affiliations:** 1 Servicio de Microbiología, Hospital Universitario Central de Asturias, Oviedo, Spain; 2 Unidad de ITS, Hospital Monte Naranco, Oviedo, Spain; 3 Servicio de Microbiología, Complejo Hospitalario Universitario de Vigo, Vigo, Spain; IPO, Inst Port Oncology, Portugal

## Abstract

**Objective:**

Human Papillomavirus (HPV) infection in men may produce cancer and other major disorders. Men play an important role in the transmission of the virus and act as a reservoir. The aim of this study was to determine the HPV-genotypes and their prevalence in a group of men attending a Sexually Transmitted Infection service.

**Patients and Samples:**

Between July 2002 and June 2011, 1392 balanopreputial, 435 urethral, 123 anal, and 67 condyloma lesions from 1551 men with a mean age of 35.8±11.3 years old (range: 17–87) were collected for HPV-DNA testing.

**Methods:**

A fragment of the L1-gene and a fragment of the E6/E7-genes were amplified by PCR. Positive samples were typed by hybridization.

**Results:**

The HPV genome was detected in 36.9% (486/1318) balanopreputial and in 24.9% (101/405) urethral (p<0.0001) swabs from 38.1% (538) of 1469 men. Co-infections were present in 5.4% (80/1469) of cases. HPV was found in 43.9% (373/850) of men younger than 35 vs. 31.7% (187/589) of men aged >35. HPV was found in 59.4% (104) of 165 men with lesions (macroscopic or positive peniscopy), and in 22.8% (61/267) without clinical alterations. HPV was also detected in 71.4% (40/56) men with condylomata and in 58.7% (64/109) of men with positive peniscopy.

**Conclusions:**

HPV prevalence in men was high and decreased with age. HPV was found more frequently in balanopreputial than in urethral swabs. There was a low rate of co-infections. Low-risk HPV vaccine genotypes were the most recurrent especially in younger. Although HPV has been associated with clinical alterations, it was also found in men without any clinical presentation. Inclusion of men in the national HPV vaccination program may reduce their burden of HPV-related disease and reduce transmission of the virus to non-vaccinated women.

## Introduction

Papillomaviruses are the most sexually transmitted viruses. Experimental and epidemiological data imply a causative role for HPVs and they appear to be the second most important risk factor for cancer development in humans, exceeded only by tobacco usage [1].

The most common cause of mortality related to human papillomavirus (HPV) infection is cervical cancer (annually 530.00 cases, with 270.000 deaths). HPV infection is also an important concern for men, due to disease burden and the risk of transmission. HPV is associated with anal cancer (approximately 90%) and a subset of penile (50%) and oral cancers (10–72%) [2], [3]. The incidence of HPV-related anal and oral cancers is increasing among the general population and it is growing even faster among immunocompromised individuals because of human immunodeficiency virus (HIV) infection [4]. Even though anal cancer is more prevalent in females than males, the risk of anal cancer in men who have sex with men (MSM) is higher [5]. The incidence of anal cancer among MSM in Australia can be estimated to be similar to that of cervical cancer before the cervical cancer screening programme was introduced. MSM are also at increased risk of developing cancers in sites that are associated with HPV in the oral cavity and oropharynx compared with the other men [6], [7]. Penile HPV infection is very common among men and remains high throughout a wide range of ages [8]–[12]. Most penile lesions are subclinical and the high prevalence of high-risk HPV suggests that they constitute a reservoir for high-risk HPV [1], [13]. HPV-16 especially, and HPV-18 are responsible for a large proportion of genital cancer [1], [14]–[17]. Other HPV-related diseases of clinical importance in men include condylomata acuminata (genital warts) and recurrent respiratory papillomatosis, which HPV-6 and HPV-11 are broadly implicated [17], [18]. Thus, knowledge and control of the human papilloma virus has a significant impact on these diseases. Prophylactic vaccination targeting these high risk genotypes is therefore expected to have a major impact on the burden of cervical cancer as well as that of other HPV-related cancers [19]. Success of HPV vaccination is now a matter of coverage [20].

The aims of this study were: 1) to assess the prevalence of Papillomavirus infection and the circulating genotypes in men who attended a Sexually Transmitted Disease service by examining urethral and paraurethral swabs; 2) to analyze the variables which may play a role in HPV acquisition and persistence; 3) and to determine which would be the best sample to perform this kind of diagnosis; 4) determine the percentages of HPV-infections in men that may be prevented by the bivalent and quadrivalent HPV vaccine.

## Results

### Incidence and sample types

**Figure 1:**
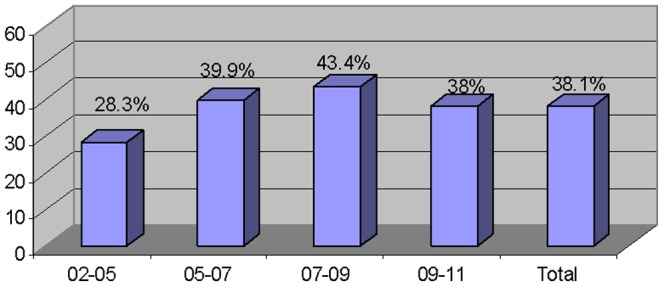
HPV incidence in males per years of study.

The HPV genome was detected in 36.9% (486/1318) balanopreputial swabs, and in 24.9% (101/405) urethral swabs (p<0.0001) from 38.1% (560) of 1469 men. In 5.4% (80/1469) of males a mixed infection was found. The incidence of HPV infection found throughout the study period was studied (figure 1). It becomes apparent that incidence increased since the beginning of the HPV infection study, reaching 43% in the 2007–2009 period; afterwards, a slight decrease was found.

In addition, HPV was present in 50.9% (34/67) lesion swabs, and in 49.6% (61/123) anal swabs (p = no significant).

### Age

**Figure 2:**
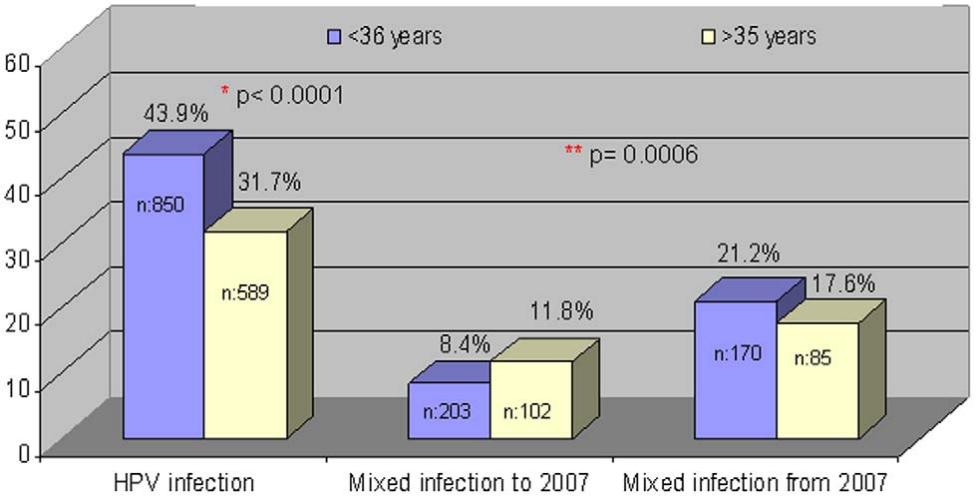
HPV and mixed infection detection rates according to male age groups and period of study.

If HPV infection is analyzed according to age, HPV was found in 48.7% (98/201) individuals younger than 25, in 42.4% (275/649) of individuals in the 25–35 range, in 31.7% (111/350) in the 36–45 range, and in 31.8% (76/239) individuals over 45 (p<0.0001). Mixed infections were detected in 9.2% (9/98) men younger than 25, in 16% (44/275) men in the 25–35 range, in 13.5% (15/111) men in the 35–45 range and in 15.8% (12/76) men over 45 years (figure 2, table S1).

On the other hand in figure 2 can observe an increase of mixed infection (co-infection) after 2007 (9.5% versus 20%) (p = 0.0006).

### Genotypes

**Figure 3:**
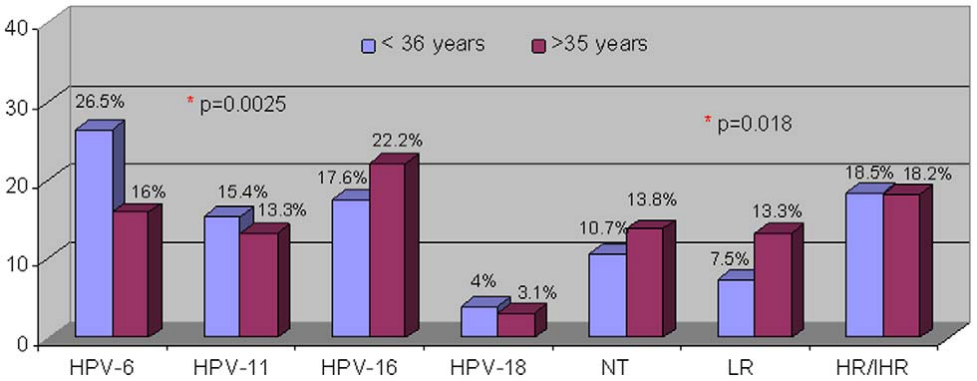
Detected genotype distribution according to age.

Low-risk genotypes (HPV-6, HPV-11, or the absence of the E6/E7 fragment) were detected in 47.1% (317/674) of patients, as opposed to 41.2% (278/674) of individuals who exhibited high-risk genotypes. High-risk genotypes are more frequent in men younger than 25 years old. However the most frecuent genotype in men younger than 25 was HPV 6 (p = 0.0013) .The involved genotype distribution by age groups was analyzed (figure 3, table 1).

**Table 1:** Distribution of vaccinal and non-vaccinal genotypes according to age.

	<36 years	>35 years	TOTAL	p
Number of HPV	449	225	674	
**VACCINAL**	**284 (63.3%)**	**123 (54.7%)**	**407 (60.4%)**	
**LR**				
HPV-6	119 (26.5%)	36 (16%)	155 (23%)	0.0025
HPV-11	68 (15.4%)	30 (13.3%)	98 (14.5%)	n.s
**HR**				
HPV-16	79 (17.6%)	50 (22.2%)	129 (19.1%)	n.s
HPV-18	18 (4%)	7 (3.1%)	25 (3.7%)	n.s
**NON-VACCINAL**	**165 (36.7%)**	**102 (45.3%)**	**267 (39.6%)**	
**NT**	*48 (10.7%)*	*31 (13.8%)*	*79 (11.7%)*	n.s
**LR**	*34 (7.5%)*	*30 (13.3%)*	*64 (9.5%)*	0.018
**HR/lHR**				
HPV-31	16 (3.6%)	8 (3.5%)	24 (3.6%)	n.s
HPV-33	11 (2.4%)	5 (2.2%)	16 (2.4%)	n.s
HPV-35	4 (1.8%)*	2 (1.9%)**	6 (1.8%)	n.s
HPV-45	6 (1.3%)		6 (1.3%)	
HPV-52	8 (3.6%)*	1 (0.9%)**	9 (2.7%)	n.s
HPV-58	18 (4%)	13 (5.8%)	31 (4.6%)	n.s
HPV-66	6 (2.7%)*	4 (3.7%)**	10 (3.1%)	n.s
HPV-NT	14 (3.1%)	8 (3.6%)	22 (8.3%)	n.s

* **n:220.**

** **n:108.**

**LR:** Low risk **HR:** High risk **lHR:** likely High risk **NT:** Non-typed.

Vaccinal genotypes, notably those with low oncogenic risk were more frequent than non-vaccinal types, except in men older than 45 years old.

As for mixed infections, table S1 shows the genotype distribution according to age groups and table S2 distribution to and from 2007, according number of probes were tested by hybridization assays.

The mixed infection rate of positive samples was 14.3% (80/560) and was not related with age (table S1). To 2007 the mixed infection rate was 9.5% (29/305), from 2007 it was 20% (51/255) (p = 0.0006) (table S2). In both periods high-risk genotypes participate in 97.5% (78/80) of the total coinfections, and HPV-16 is the most frequent one, having been detected in 67.5% (54/80) of cases, followed by HPV-6, found in 42.5% (34/80) coinfections, and HPV-11, which was detected in 25% (20/80) of subjects. The incidence rate of the remaining genotypes was lower.

On the other hand, 15.9% (89/560) of patients presented HPV-16 and/or HPV-18 without other genotypes. And 40% (224/560) presented HPV-6 and/or HPV-11 and/or HPV-16 and/or HPV-18 without other genotypes.

### Clinical presentation

The relationship between HPV infection and several factors associated to it or its progression is shown in table 2.

**Table 2:** Association of the different factors with HPV infection in males. HPV +/− (%).

Infection factors (HPV+)	YES	NO	p
**PDA**	4/8 (33.3)	158/264 (37.4%)	ns
**STD**	89/86 (50.8%)	133/138 (49%)	ns
**Promiscuity**	86/164 (34.4%)	97/157 (38.2%)	ns
**Marital Status**			
Single	119/179 (39.9%)		
Divorced	17/26 (39.5%)		0.05
Married	35/112 (23.8%)		

Progression factors (E6/E7+)	YES	NO	
**Alcohol**	32/62 (34%)	20/43 (31.7%)	ns
**Tobacco usage**	28/70 (28.6%)	24/36 (40%)	ns

**PDA:** Parenteral drug addiction; **STD:** Sexually transmitted disease.

The results of macroscopic penis examinations were known for 151 men; 56 featured alterations compatible with condylomata at the time of exploration, and HPV was detected in 71,4% (40/56); however, only 16,8% (16/95) out of males with negative exploration were HPV positive (p<0.0001). After performing a peniscopy, HPV was present in 58.7% (64) out of 109 patients with lesions compared to 26.6% (45) of 172 patients without lesions (p<0.0001).

Of all the studied variables, only marital status was related to the infection: single and divorced men have a higher viral infection rate than married men (p = 0.05).

## Discussion

While much is known about the natural history of cervical HPV infection and its consequences, including cervical intraepithelial neoplasia and cervical cancer, relatively little is known about the natural history of genital HPV infection and diseases in men. In part this reflects difficulties in penile sampling and visual assessment of penile lesions. Although HPV is transmitted sexually and infects the genitals of both sexes, the cervix remains biologically more vulnerable to malignant transformation than the penis or anus in men. However, as more precise and sensitive methods become available and wider studies are performed, the number of HPV-associated pathologies has also increased [22]–[24]. Understanding male HPV infection is therefore important in order to reduce HPV pathology and its transmission [25]–[27].

The HPV infection rate increased steadily throughout the years of the study in the male population in our area. In the first years of the study (2002–2005), it was 28.3% to 43% in 2007–2009. This rate remained stable in the latest years after solving some issues related to sample types and sample gathering, and improving the assessment of individuals with a potential pathology. The 36.3% incidence rate in men attending a STI clinic is close to the highest values described [5]–[12], [28]; hence, we can state that men constitute a critical link in the transmission of HPV infection [27], especially if those men have multiple sexual partners and are in contact with risk groups [29], [30].

Data on infection incidence take into account the results of the urethral and paraurethral swabs. As other authors have already described, paraurethral swabs exhibited a greater diagnostic performance, which facilitates the performance of this type of studies, since these samples can be easily obtained [5], [10], [31]–[33].

In this study, swabs obtained from anal lesions or penile condylomata were also processed. The performance of these types of samples was lower than expected (50%), but it must be taken into account that no biopsies were carried out. Biopsies would be the preferred samples to perform an etiologic diagnosis of lesion samples [34].

No evident influence of age on infection rate has been found in the published literature on HPV infection in men [6], [35]. In the U.S., Stone *et al.* [36] found a higher prevalence rate in the 30–39 age range. Our series, however, reveal a decrease in the infection rate as age increases, just as described for the female population [37]–[39]; however, the percentages are always higher than those found in women, in any age range [40].

The more frequent viral genotypes in the male population were the vaccinal genotypes, notably HPV-6 and HPV-11, a fact already mentioned by other authors [11], [29]. In this study, low-risk oncogenic genotypes were found with a 47% frequency, a value that contrasts with other publications in which LR genotype prevalence was between 2.3% and 24%. Further more, HPV 6 was found more frequently in men younger than 35 years old. As opposed to the findings of most other studies in which the HPV-16 is the most frequent genotype [10], [41]. In this series HPV-16 was only more frequent in men older than 35 but not significantly. A possible explanation of this fact would be that many of these men featured subclinical lesions, all caused by low-risk HPV (6 and 11), as revealed by peniscopy. In keeping with this, Partridge and Koustky [29] concluded that the epithelium of the penis seems to be less receptive to high-risk genotypes.

According to these results, male vaccination with the tetravalent vaccine would be more effective than with the bivalent one, since it could reduce the risk of anal-genital warts, which, although not a severe pathology, do cause significant psychological morbility.

In the present study, to 2007 a 3.1% of mixed infections were detected. The prevalence of multiple-type HPV has been reported to be between 2.1% and 34.8% in men [10], [12], [42]–[44]. Technical limitations may condition the results. However, a large-size fragment amplification was carried out, and the genotyping was performed by hybridization techniques which allow for the detection of minuscule populations more efficiently than sequencing techniques, although the number of probes was limited to the most common genotypes found in our environment. This limited amount of probes may underestimate the amount of mixed infections with uncommon genotypes. From 2007, when seven probes were added, the prevalence of multiple-type HPV raises 20%. The 97.5% mixed infections were composed by high-risk genotypes, but only less than half of the cases (38.8%) corresponded to the genotypes included in the FDA-approved quadrivalent vaccine for men [45].

The individuals from this study that would have benefit by the bivalent vaccine will be 15.9% and by the quadrivalent vaccine 40%.

The virus was more frequently detected in those men showing a clinical presentation compatible with HPV infection than in those who did not. In these cases, however, the percentage of HPV found was not higher than 51%. Obtaining cellular samples by exfoliation of the keratinized epithelium was difficult in those instances, thus compromising the diagnosis [46].

The risk of contagion is related to sexual behaviour (number of sexual partners, both throughout life and simultaneous), sexual intercourse with risk groups (prostitution), polygamy, having suffered other STDs and parenteral drug use. In our study, none of these factors seemed to be involved in the increase of the HPV infection rate, with the exception of marital status: single or divorced individuals were associated to a higher HPV infection rate. These males also exhibit a sexual behaviour different to that of married men.

Although many males get infected with HPV, most of such infections do not develop into cancer. It is thus probable that other co-factors, such as tobacco usage, alcohol intake and coinfection with other sexually transmitted diseases play a role in the pathologic process. No relationship between these co-factors and the presence of oncogenic genotypes was established in this study.

On this study, urethral and paraurethral swabs are not collection simultaneously, and clinical and pathological data were reviewed only from 151 patients due to the medical history confidentiality. These limitations could have any influence in the data.

To sum up, although HPV infection do have worse consequences for women, men play an active role in it, as the high incidence rates found demonstrate. Men are involved in its reservoir and transmission, and the virus may end up causing major disorders and severe consequences. The high prevalence in this collective suggests the need of more exhaustive assessments, even though most individuals do not feature high-risk oncogenic viruses. Most countries have only included girls in their national vaccinations programs [47], and MSM will not be indirectly protect. Inclusion of men in the national HPV vaccination program may reduce their burden of HPV-related disease and reduce transmission of the virus to non-vaccinated women.

## Materials and Methods

### Patients

Between June 2002 to June 2011, HPV genome detection was performed prospectively in 1551 males (mean age: 35.8±11.38 years old, range: 17–87 years old) which were attended in the Sexually-transmitted Infections Service of the Hospital Monte Naranco (Oviedo, Asturias; North of Spain). From them 1318 paraurethral swabs, 405 urethral swabs, 123 anal and 67 condyloma lesions were recovered.

Clinical data together with HPV infection-related risk factors (marital status, a history of STDs, alcohol intake, tobacco usage, and parenteral drug abuse) were gathered from 515 patients. From them, in 151 macroscopic penis data, and in 286 peniscopy data were also recovered.

### Methods

#### DNA Extraction

Half the volume of the samples was homogenized and concentrated by centrifugation at 6000 rpm (1800 g) for 5 min and supernatant removed. Then, 200 µl of lysis solution (10 mM Tris-HCL pH 8.3, 50 mM potassium chloride, 2.5 mM magnesium chloride, 0.5% Igepal, 0.5% Tween 20 and 10 µg of proteinase K) was added, mixed and incubated for 45 min at 56°C, then for 10 min at 96°C to inactivate proteinase K, and finally stored at −20°C until it was analyzed. The quality of extracted DNA was checked by PCR amplification of the β-globin gene (the primer forward 5′-ACACAACTTGTGTGTTCACTAGC-3′and reverse 5′-CAAACTTCATCCACGTTCACC-3′). If there were problems with PCR, an automated extraction with a TNAI kit (Roche Diagnostic, S.L.; Spain) from stored samples was performed. From 2009, in all the samples automated extraction was used.

#### DNA amplification (PCR assay)

Standard protocol of PCR with MY11/MY09 primers (site-directed L1 fragment of HPV) [21] was developed with all specimens in order to detect a broad spectrum of HPV genotypes, as described previously. Briefly, the PCR was performed in 50 µl of reaction mixture containing 1× PCR buffer, 2 mmol/L MgCl2, 50 µmol/L of each deoxynucleoside, 0.5 µmol/L of sense and antisense primer, 10 µl of sample and 1 U Taq DNA polymerase (Bioline, USA), by thermal profile of 35 cycles: denaturation at 94°C for 30 sec, annealing at 55°C for 30 sec and extension at 72°C for 1 min, with an initial denaturation at 94°C for 5 min and a final extension at 72°C for 10 min. The amplified DNA fragments of approximately 450 bp were identified by electrophoresis in 1.5% agarose gel with ethidium bromide. From 2009, GP5+/GP6+ primers (site-directed L1 fragment of HPV) also were incluyed in PCR was used with the same protocol.

Another PCR was performed with a pair of primers against the E6/E7 fragment (onco1-sense- 5′-TGTCAAAAACCGTTGTGTCC-3′, and onco2- antisense-5′-GAGCTGTCGCTTAATTGCTC-3′). PCR was done in 25 µl of reaction mixture containing 1× PCR buffer, 2 mmol/L MgCl2, 50 µmol/L of each deoxynucleoside, 0.5 µmol/L of sense and antisense primer, 10 µl of DNA template and 1 U Taq DNA polymerase (Bioline, USA) by thermal profile at 40 cycles: 95°C for 30 sec, 55°C for 30 sec, and 72°C for 30 sec. This was followed by a final extension at 72°C for 5 min. An initial denaturation at 95°C during 5 min was also carried out. Products about 250 bp in sizes were expected.

Positive specimens from different HPV types were used as positive controls. The reaction mixtures containing no DNA, water, and DNA from human leucocytes were used as negative controls.

#### DNA hybridization

All positive specimens for L1 fragment were tested by hybridization assays using type-specific probes for HPV types 6 (MY12: 5′-CATCCGTAACTACATCTTCCA-3′), HPV-11 (MY13: 5′-TCTGTGTCTAAATCTGCTACA-3′), HPV-16 (MY14: 5′-CATACACCTCCAGCACCTAA-3′), HPV-18 (WD74: 5′-GGATGCTGCACCGGCTGA-3′), HPV-31 (WDB128: 5′-TTGCAAACAGTGATACTACATT-3′), HPV-33 (MY16: 5′-CACACAAGTAACTAGTGACAG-3′), HPV-45 (MYB69: 5′-ATACTACACCTCCAGAAAAGC-3′) as previously described by Ting and Manos^22^, and an “in-house” design probe against HPV-58 (SANTI58: 5′-TGAAGTAACTAAGGAAGGTACA-3′).

From 2007 were added probes against HPV types 35 (5′-CTGCTGTGTCTTCTAGTGA-3′), HPV- 39 (5′-ATAGAGTCTTCCATACCTTC-3′), HPV- 51 (5′-TGCTGCGGTTTCCCCAA-3′), HPV- 52 (5′-GAATACCTTCGTCATGGC-3′), HPV- 56 (5′-TGTCTACATATAATTCAAAGC-3′), HPV- 66(5′-AGCTAAAAGCACATTAACTAA-3′), HPV- 68 (5′-CTGAATCAGCTGTACCAAT-3′).

#### Data and statistical analysis

To avoid prevalence bias, only the first specimen positive for HPV DNA from a given sample was considered Prevalence rates of four age groups were used. Statistical tests (Chi-square test, Fisher's test, Student's t-test, etc.) were performed using GraphPad InStat version 3.00 for Windows 95 (GraphPad Software, San Diego, CA) following the manufacturer's instructions.

All experiments were performed in compliance with relevant laws and institutional guidelines and in accordance with the ethical standards of the Declaration of Helsinki. Information concerning the research project was provided to all participants, and all signed a free and informed consent from approved by Ethical committee of Clinical Investigation of the HUCA.

## Supporting Information

Table S1 **Distribution of patient detected genotypes in mixed infections according to age.**
(DOC)

Table S2 **Distribution of patient detected genotypes in both periods of study.**
(DOC)
