# Supplementary material for: Human Papillomavirus Infection in a Male Population Attending a Sexually Transmitted Infection Service
Source: PLoS One. 2013 Jan 23;8(1):e54375. doi: 10.1371/journal.pone.0054375 (PMC3553085; doi:10.1371/journal.pone.0054375)
Supplement: Table S1 — Distribution of patient detected genotypes in mixed infections according to age. (DOC) [file pone.0054375.s001.doc]

| **Table** **S1.** Distribution of patient detected genotypes in mixed infections according to age. | | | |
| --- | --- | --- | --- |
|  | **<36 years** | **>35 years** | **TOTAL (%)** |
| **Mixed infections** | **53/373 (14.2%)** | **27/187 (14.4%)** | **80/560 (14.3%)** |
| **LR** | *2* |  | *2 (0.4%)* |
| HPV-6/11 | 2 |  | 2 |
| **LR-HR/lHR** | *33* | *16* | *49 (8.8%)* |
| HPV-6/11/16 |  | 1 | 1 |
| HPV-6/11/16/66 |  | 1 | 1 |
| HPV-6/11/18 | 1 |  | 1 |
| HPV-6/16 | 8 | 3 | 11 |
| HPV-6/16/31 | 1 |  | 1 |
| HPV-6/16/33 | 1 |  | 1 |
| HPV-6/16/52 | 1 |  | 1 |
| HPV-6/16/53/66 | 1 |  | 1 |
| HPV-6/16/58 | 1 |  | 1 |
| HPV-6/16/31/58 | 1 |  | 1 |
| HPV-6/16/18 | 1 |  | 1 |
| HPV-6/18 | 1 |  | 1 |
| HPV-6/18/31 |  | 1 | 1 |
| HPV-6/18/45/58 | 1 |  | 1 |
| HPV-6/31 | 1 |  | 1 |
| HPV-6/33 | 1 | 1 | 2 |
| HPV-6/58 | 3 | 1 | 4 |
| HPV-6/66 | 1 |  | 1 |
| HPV-11/16 | 4 | 4 | 8 |
| HPV-11/18 | 2 |  | 2 |
| HPV-11/31 |  | 1 | 1 |
| HPV-11/33 | 1 |  | 1 |
| HPV-11/35 | 1 |  | 1 |
| HPV-11/16/31 | 1 |  | 1 |
| HPV-11/16/33 |  | 1 | 1 |
| HPV-11/58 |  | 2 | 2 |
| **HR/lHR** | *19* | *10* | *29 (5.2%)* |
| HPV-16/18 | 3 | 1 | 4 |
| HPV-16/18/35/58 |  | 1 | 1 |
| HPV-16/18/58 | 1 |  | 1 |
| HPV-16/18/66 |  | 1 | 1 |
| HPV-16/31 | 2 |  | 2 |
| HPV-16/31/35/52/58 | 2 |  | 2 |
| HPV-16/33 | 1 | 1 | 2 |
| HPV-16/33/39 | 1 |  | 1 |
| HPV-16/33/58 |  | 1 | 1 |
| HPV-16/35/52/58 |  | 1 | 1 |
| HPV-16/35/58 | 1 |  | 1 |
| HPV-16/45 | 1 |  | 1 |
| HPV-16/52 | 1 |  | 1 |
| HPV-16/53/66 | 1 |  | 1 |
| HPV-16/58 |  | 1 | 1 |
| HPV-16/59 | 1 | 1 | 2 |
| HPV-16/66 |  | 1 | 1 |
| HPV-18/52/66 | 1 |  | 1 |
| HPV-31/33 | 1 |  | 1 |
| HPV-31/45/52 | 1 |  | 1 |
| HPV-35/58 |  | 1 | 1 |
| HPV-58/66 | 1 |  | 1 |
| **LR:** Low risk **HR:** High risk **lHR:** likely High risk **NT:** Non-typed | | |  |
