# Supplementary material for: Human Papillomavirus Infection in a Male Population Attending a Sexually Transmitted Infection Service
Source: PLoS One. 2013 Jan 23;8(1):e54375. doi: 10.1371/journal.pone.0054375 (PMC3553085; doi:10.1371/journal.pone.0054375)
Supplement: Table S2 — Distribution of patient detected genotypes in both periods of study. (DOC) [file pone.0054375.s002.doc]

| **Table S2.** Distribution of patient detected genotypes in both periods of study. | | | | | |
| --- | --- | --- | --- | --- | --- |
|  |  | **2002-2007** | **2007-2011** | **TOTAL (%)** |  |
|  | **Mixed infections** | **29/305 (9.5%)** | **51/255 (20%)** | **80/560 (14.3%)** |  |
|  | **LR** | ***1 (0.3%)*** | *1 (0.4%)* | *2 (0.4%)* |  |
|  | HPV-6/11 | 1 | 1 | 2 |  |
|  | **LR-HR/lHR** | *23 (7.5%)* | *26 (10.2%)* | *49 (8.8%)* |  |
|  | HPV-6/11/16 |  | 1 | 1 |  |
|  | HPV-6/11/16/66 |  | 1 | 1 |  |
|  | HPV-6/11/18 | 1 |  | 1 |  |
|  | HPV-6/16 | 7 | 4 | 11 |  |
|  | HPV-6/16/31 |  | 1 | 1 |  |
|  | HPV-6/16/33 | 1 |  | 1 |  |
|  | HPV-6/16/52 |  | 1 | 1 |  |
|  | HPV-6/16/53/66 |  | 1 | 1 |  |
|  | HPV-6/16/58 |  | 1 | 1 |  |
|  | HPV-6/16/31/58 | 1 |  | 1 |  |
|  | HPV-6/16/18 | 1 |  | 1 |  |
|  | HPV-6/18 |  | 1 | 1 |  |
|  | HPV-6/18/31 |  | 1 | 1 |  |
|  | HPV-6/18/45/58 |  | 1 | 1 |  |
|  | HPV-6/31 | 1 |  | 1 |  |
|  | HPV-6/33 | 2 |  | 2 |  |
|  | HPV-6/58 | 1 | 3 | 4 |  |
|  | HPV-6/66 |  | 1 | 1 |  |
|  | HPV-11/16 | 4 | 4 | 8 |  |
|  | HPV-11/18 | 1 | 1 | 2 |  |
|  | HPV-11/31 |  | 1 | 1 |  |
|  | HPV-11/33 |  | 1 | 1 |  |
|  | HPV-11/35 |  | 1 | 1 |  |
|  | HPV-11/16/31 | 1 |  | 1 |  |
|  | HPV-11/16/33 | 1 |  | 1 |  |
|  | HPV-11/58 | 1 | 1 | 2 |  |
|  | **HR/lHR** | *5 (1.6%)* | *24 (9.4%)* | *29 (5.2%)* |  |
|  | HPV-16/18 | 2 | 2 | 4 |  |
|  | HPV-16/18/35/58 |  | 1 | 1 |  |
|  | HPV-16/18/58 |  | 1 | 1 |  |
|  | HPV-16/18/66 |  | 1 | 1 |  |
|  | HPV-16/31 | 1 | 1 | 2 |  |
|  | HPV-16/31/35/52/58 |  | 2 | 2 |  |
|  | HPV-16/33 |  | 2 | 2 |  |
|  | HPV-16/33/39 |  | 1 | 1 |  |
|  | HPV-16/33/58 |  | 1 | 1 |  |
|  | HPV-16/35/52/58 |  | 1 | 1 |  |
|  | HPV-16/35/58 |  | 1 | 1 |  |
|  | HPV-16/45 |  | 1 | 1 |  |
|  | HPV-16/52 |  | 1 | 1 |  |
|  | HPV-16/53/66 |  | 1 | 1 |  |
|  | HPV-16/58 | 1 |  | 1 |  |
|  | HPV-16/59 |  | 2 | 2 |  |
|  | HPV-16/66 |  | 1 | 1 |  |
|  | HPV-18/52/66 |  | 1 | 1 |  |
|  | HPV-31/33 | 1 |  | 1 |  |
|  | HPV-31/45/52 |  | 1 | 1 |  |
|  | HPV-35/58 |  | 1 | 1 |  |
|  | HPV-58/66 |  | 1 | 1 |  |
|  | **LR:** Low risk **HR:** High risk **lHR:** likely High risk **NT:** Non-typed | | |  |  |
